# Fluid balance and mortality in critically ILL patients

**DOI:** 10.1186/2197-425X-3-S1-A532

**Published:** 2015-10-01

**Authors:** Y Ben Aicha, A Ben Souissi, S Kamoun, S Koubaji, F Haddad, MS Mebazaa

**Affiliations:** Anesthesiology and ICU Department, Mongi Slim University Hospital La Marsa, Sidi Daoued, Tunisia

## Introduction

Intravenous fluids are widely administrated to correct intravascular volume deficit or acute hypovolemia. However, generalized endothelial injury and capillary leak is a common condition in critically ill patients. In such a state, overzealous fluid therapy results in extravascular movement of water, electrolytes and proteins leading to tissue edema and organ dysfunction. The aim of this study is to assess whether positive fluid balance is associated with worse outcomes and increased mortality in critically ill patients.

## Methods

This was a retrospective study. All patients admitted between 1 July and 31 December 2014 were eligible for the study, except those who stayed in the ICU for less than 48 hours. We collected data on demographics, comorbidity conditions, APACHE II and SAPSII scores. Acute kidney injury was classified using the worst RIFLE score obtained during the first 7 days and the need of renal replacement therapy. Vasopressors use, ICU mortality, hospital lengths of stay and mechanical ventilation were also registered. The fluid balances were calculated at the second, 7, 14, 21 day and at the hospital discharge.

## Results

A total of 95 patients were included to the cohort study. Septic shock represented 66% of the cases. Median age was 53 ± 19 years old. 64% were men. The mean APACHE II score was 20 ± 7, and the mean SAPSII score was 46 ± 18. The mortality rate was 37.5%.

We examined cumulative fluid balance among patients who died and those that survived (table1).

**Table 1:** *Cumulative fluid balance (mL).*

	Survivors (n = 56)	Non-Survivors (n = 39)	p value
Day 2	2120 ± 1670	2839 ± 1235	0.023
Day 7	5763 ± 2311	6947 ± 3005	0.019
Day 14	4024 ± 1986	4673 ± 2265	0.031
Day 21	4542 ± 2567	7243 ± 3480	0.023
Total fluid balance	5882 ± 3887	10949 ± 4564	0.019

To evaluate the relationship between cumulative fluid balance and outcome we stratified our study population into 4 groups based upon their cumulative balances observed on hospital discharge: less than zero (n = 2), 1 to 5 L positive balance (n = 5), 5 to 10 L positive balance (n = 13), and over 10 L positive balance (n = 19). Among these 4 fluid balance groups, those who had negative or less positive fluid balance had lower mortality compared to those with the larger positive fluid balances.

The need for renal replacement therapy, and RIFLE score of 3 were more frequent among non-survivors (p < 0.05).

Non-survivors had also significantly fewer ICU hospital days (14 days vs. 19 days p = 0.045) and markedly more days free from mechanical ventilation (6 days vs. 14 days p = 0.036). The use of vasopressors was also more prolonged among the same group (p = 0.034).

## Conclusions

Positive cumulative fluid balance is associated with significantly higher mortality and worse outcomes. Our results raise the hypothesis that a judicious fluid balance after early resuscitation might be a useful tool to improve prognosis in critically ill patients.

